# The feasibility of *Sorghum* (*Sorghum vulgare*) fodder as poultry feed ingredients seen from growth performance, nutrient content and fiber profile of *Sorghum* fodder

**DOI:** 10.5455/javar.2023.j672

**Published:** 2023-06-30

**Authors:** Cahya Setya Utama, Bambang Sulistiyanto, Muhammad Fikri Haidar

**Affiliations:** Faculty of Animal and Agricultural Sciences, Diponegoro University, Semarang, Indonesia

**Keywords:** Feed, fodder, *Sorghum*, proximate, SEM-EDX

## Abstract

**Objectives:**

The aim of the study was to assess the feasibility of fodder *Sorghum* as poultry feed in terms of growth performance (plant height and fresh weight), nutritional quality (moisture, ash, crude protein, extract ether, crude fiber, extract material without nitrogen, and metabolic energy), and scanning electron microscopy energy dispersive X-ray (SEM-EDX).

**Materials and Methods:**

The study used a completely randomized design with six treatments and three replications. The treatments consisted of planting times of 24, 48, 72, 120, and 240 h and a control (0 h).

**Results:**

The results showed that there was a significant effect (*p* ≤ 0.05) when planting *Sorghum* fodder (SGF) on growth performance and moisture, but it had no significant effect on fresh weight, ash, crude protein, extract ether, crude fiber, nitrogen-free extract, energy metabolic aspects, and SEM-EDX.

**Conclusion:**

SGF is suitable as a feed ingredient for poultry in terms of nutrition and contains ZrO_2_, which functions as an antifungal.

## Introduction

Fodder is a plant or forage that can be used as feed, which is grown in a short time. The fodder method is done by sowing grains such as corn, *Sorghum*, and wheat in a medium [1]. *Sorghum* has the potential to be developed as green fodder because it can thrive in a tropical environment and is efficient in its maintenance process [2]. *Sorghum* fodder (SGF) contains 15.41% crude protein, 8.44% extract ether, and 11,03% crude fiber [3]. Fodder is suitable for use as poultry feed because of its low crude fiber content and reduced tannin content [4]. *Sorghum* cultivation can be done using a fodder system to reduce its anti-nutritional content [5]. The low crude fiber content of 2%–5% fodder has an impact on the increased digestibility of the material. The age of the plants, humidity levels, light, temperature, and media conditions all have an impact on the cultivation of fodder. The relatively short harvesting age makes fodder one of the solutions for the shortage of feed ingredients [6].

SGF can be used as an alternative source of energy for poultry. The use of fodder as broiler chicken feed can increase carcass weight. Colă and Colă [7] reported that using 23% fodder in broiler feed increased the carcass weight of broiler chickens by about 5%–10%. Based on the research of Aqilla et al. [8], the use of fodder as a hybrid chicken feed can increase egg production by 19.75%. Saputra et al. [9] added that using fodder with a composition of 6%–9% in hybrid chickens can increase the index of egg shape, fertility, hatchability, and hatching weight of chickens. The advantages of fodder are its relatively short planting time, good nutritional and digestibility content, and reduced anti-nutritional content [10]. The fodder planting system is mostly applied to corn plants. This study used *Sorghum* with a fodder planting system because of its large availability and good nutritional content. The purpose of the study was to assess the feasibility of fodder *Sorghum* as poultry feed in terms of growth performance (plant height and fresh weight), nutritional quality [moisture, ash, crude protein, extract ether, crude fiber, nitrogen-free extract (NFE), and energy metabolism (EM)], and scanning electron microscopy energy-dispersive X-ray (SEM-EDX). The benefit of the research is to provide information about the use of SGF as poultry feed. The research hypothesis is that fodder *Sorghum* can be used as feed for poultry.

## Materials and Methods

The materials used were white *Sorghum* (*Sorghum vulgare*) from the local market, an analytical balance kern ABJ-220 with an accuracy of 0.001 gm, a 30 × 20 cm tray, a Universal Drying Oven UN 55, a Thermo F48010-26 furnace, a Normax Portugal desiccator, a Boro3.3 Germany glass beaker, a 50-ml Erlenmeyer, a filter paper, a Whatman filter paper, a Kjeldahl flask, a filter flask, an aluminum foil, and a Soxhlet Type II Flask /1-4360-04.

The research used a complete randomized design with six treatments and three repeats. The treatments were as follows:

T0: Planting age 0 h (control)

T1: Planting age 24 h

T2: Planting age 48 h

T3: Planting age 72 h

T4: Planting age 120 h

T5: Planting age 240 h

Planting of SGF begins with the cleaning of 1 kg of *Sorghum* seeds for each treatment. The clean seeds were then lowered using water at 100°C for 24 h, and then the seeds were spread on the planting medium according to the treatment. Watering is done twice a day, in the morning and evening. Parameters observed were growth performance (plant height and fresh weight), nutrient content (moisture, ash, crude protein, extract ether, crude fiber, NFE, and EM), and SEM-EDX.

### Growth performance

Growth performance was tested by measuring plant height and fresh weight. The measurement of plant height was carried out before harvesting by measuring from the base of the planting medium to the top of the plant using a ruler. Fresh weight was measured during the harvesting process by weighing the yields.

### Nutritional content

Parameters of nutrient content were tested by proximate analysis according to AOAC [11], including moisture by oven at 110°C, ash content by ashing, protein content by Kjeldahl method, extract ether content by Soxhlet method, and fiber content by gravimetric method. The content of the NFE was calculated using the formula, according to the method of Traughber et al. [12], namely, NFE = 100—crude protein—extract ether—ash—crude fiber. Metabolic energy is calculated using Balton’s formula, namely, EM (kcal/kg) = 40.81 [0.87 (crude protein + 2.25 × extract ether + NFE) + 2.5] [13].

### SEM-EDX testing

SEM-EDX testing was carried out using a scanning electron microscope (SEM) (US) and energy dispersive X-ray using Fourier transform infrared spectroscopy (Perkin Elmer, US). The sample is then tested in the laboratory to determine the elemental composition with energy dispersive X-ray, according to the procedure.

### Data analysis

The data obtained were analyzed using the analysis of variance test to test data diversity and determine if there is a real influence, followed by Duncan’s multiple range test with a 95% confidence level.

## Results and Discussions

### Growth performance of SGF with different planting times

The results of the data analysis showed that the height of fodder *Sorghum* plants with different planting times showed significant differences (Table 1). The lowest plant height was at T0, with 0 cm, and the highest was at T5, with a height of 6.1 cm. The value of plant height and fresh weight of SGF increased with the increasing age of harvest. Chrisdiana [2] stated that the biomass of green fodder would increase along with the increasing age of harvest. The ratio of stems and leaves will increase so that it will increase plant height, which is a representation of plant biomass [14].

The fresh weight of SGF with different planting times did not experience any significant difference. The value of plant weight can be increased by the increased conversion of nutrients obtained from water and stored in seeds during the rearing process into plant parts. Kusdiana et al. [15] stated that fresh weight per clump is one of the parameters in the growth of a plant and also plays a role in determining the quality of yield or production, which data is taken after harvest. Green fodder plant growth is strongly influenced by the availability of nutrients in the seeds. Rousseau et al. [16] stated that plants that lack nutritional elements experience obstacles in the formation of green leaves, which play a very important role in photosynthesis so that the formation of carbohydrates that function for energy and cell formation for plant growth is reduced as a result of plants turning yellow and slow growth. Fodder *Sorghum* can produce a dry weight of about 60%–70% of its fresh weight.

**Table 1.** Growth performance of SGF with different planting time.

Parameters	Treatment					
	T0	T1	T2	T3	T4	T5
Plant height (cm)	0^a^	1.6^b^	1.9^b^	2.4^bc^	4.8^d^	6.1^d^
Fresh weight (gm)	1,097	1,166	1,187	1,156	956	946

Different superscripts on the same line show a noticeable difference (*p *< 0.05).

### Nutritional content of SGF with different planting time

The nutritional content of SGF with different sowing times is shown in Table 2.

### Moisture

Based on data analysis, the difference in planting time affects the water content of SGF. The water content of SGF in treatment T0 was 36.44%, significantly different from treatments T1, T2, T3, T4, and T5, while treatments T1, T2, T3, T4, and T5 were not significantly different between the five treatments, with a water content of around 21.60%–22.84%. Chrisdiana [2] stated that the water content of SGF ranges from 60% to 74.5%. The high water content in the T0 treatment was caused by the lack of nutrients, so the water component was still high. Wahyono et al. [4] stated that the conversion of plant nutrients would increase along with the increasing age of harvest. The moisture of SGF is influenced by differences in plant commodities, the use of nutrient solutions, and the determination of harvest age [17].

### Ash

SGF ash content at different planting times was not significantly different. The SGF in this study contained an ash content of around 0.85%–1.48%. The ash content value of hydroponic sorghum fodder with a planting time of 8 days was 2.25% [2]. The value of the ash content of a feed ingredient shows the large number of minerals contained in the feed material [18]. The low value of SGF ash content is possible because of the shorter research planting age. Soni et al. [19] stated that the age of *Sorghum* planting would affect the mineral and organic matter content.

### Crude protein

Differences in planting time did not affect the protein content of SGF. The protein content of SGF ranged from 9.43% to 10.17%. The absence of significance in the protein content is possible because the carbohydrate fraction content of the SGF is relatively the same. Pan et al. [20] stated that during germination and growth, plants use carbohydrate reserves, which are assimilated by their metabolic activities, thereby increasing the crude protein fraction. Factors that affect protein content are harvesting age, type of seed, and plant food reserves. Chrisdiana [2] reported that the longer the harvest, the higher the crude protein content of *Sorghum*.

### Extract ether

The results of the data analysis showed that the extract ether content of *Sorghum* feed was not affected by planting time. The extract ether content of *Sorghum *feed is 1.76%–3.51%. Sriagtula et al. [3] stated that the extract ether content of feed *Sorghum* extract was around 8.44%. There was no effect of differences in planting time on extract ether content due to soaking in hot water, which changed the fat fraction to free fatty acids. Chrisdiana [2] stated that soaking the seeds will increase the activity of enzymes that can convert fat into free fatty acids. The low extract ether content can minimize feed damage.

### Crude fiber

Different planting times had no impact on the crude fiber content of *Sorghum* feed. The value of fiber content was not significantly different because of the relatively short planting time. Suhartanto et al. [21] stated that in connection with the development and increasing age of plants, there would also be an increase in fiber concentration. Short planting aims to reduce the crude fiber content so that digestibility increases. In the early phase of plant growth, the development of the fiber fraction is very important to support metabolism and strengthen plant stands. Wahyono et al. [4] stated that the accumulation of an increased cell wall fraction was associated with an increase in crude fiber content with increasing harvest time.

**Table 2.** Nutritional content of SGF with different planting time.

Parameters	Treatments					
	T0	T1	T2	T3	T4	T5
Moisture (%)	36.44 ± 5.89^a^	22.30 ± 2.55^b^	21.85 ± 2.31^b^	21.68 ± 2.18^b^	21.60 ± 2.36^b^	22.84 ± 2.01^b^
Ash (%)	1.24 ± 0.22	0.85 ± 0.21	1.04 ± 0.20	1.48 ± 0.27	1.34 ± 0.22	1.32 ± 0.21
Crude protein (%)	9.43 ± 0.24	9.65 ± 0.22	9.71 ± 0.27	9.74 ± 0.28	10.17 ± 0.31	9.75 ± 0.26
Extract ether (%)	3.46 ± 0.69	2.24 ± 0.52	2.49 ± 0.61	1.76 ± 0.60	3.51 ± 0.72	2.73 ± 0.59
Crude fiber (%)	2.49 ± 0.41	2.32 ± 0.37	1.87 ± 0.32	1.26 ± 0.31	5.33 ± 0.49	2.90 ± 0.47
Nitrogen-free extract (%)	83.37 ± 2.41	84.94 ± 2.78	84.89 ± 2.83	85.75 ± 2.52	79.66 ± 2.33	83.30 ± 2.39
EM (kcal/kg)	3,673.59 ± 35.41	3,639.41 ± 35.35	3,659.63 ± 35.27	3,633.33 ± 34.87	3,571.43 ± 34.22	3,623.86 ± 35.19

Different superscripts on the same line show a noticeable difference (*p *< 0.05).

### Nitrogen-free extract

The NFE value of fodder *Sorghum* did not significantly differ between the different planting time treatments. This is because planting time has no effect on other components such as crude fiber, crude fat, and crude protein. The factors that affect the NFE value are ash, crude fiber, crude protein, and extract ether levels. Aqilla et al. [8] stated that NFE comprises carbohydrates, amino acids, and vitamins. NFE contains monosaccharides, disaccharides, trisaccharides, and polysaccharides, especially starch, which is easily soluble in acid and alkaline solutions in crude fiber analysis and has high digestibility.

### Energy metabolism

The results of the data analysis showed that the metabolic energy value of SGF was not affected by planting time. The EM value of fodder *Sorghum* ranged from 3,571.43 to 3,673.59 kcal/kg. The high and low metabolic energy content of a feed ingredient is influenced by the content of other nutrients, such as crude fiber content. According to Hidayat [22], the content of crude fiber in a material will affect the value of metabolic energy. The content of the metabolic energy value of a feed will affect the level of feed consumption. The higher the metabolic energy value, the lower the feed consumption; in addition, the metabolic energy value is also related to the digestibility value [23].

### SEM-EDX observation

**Figure 1.**
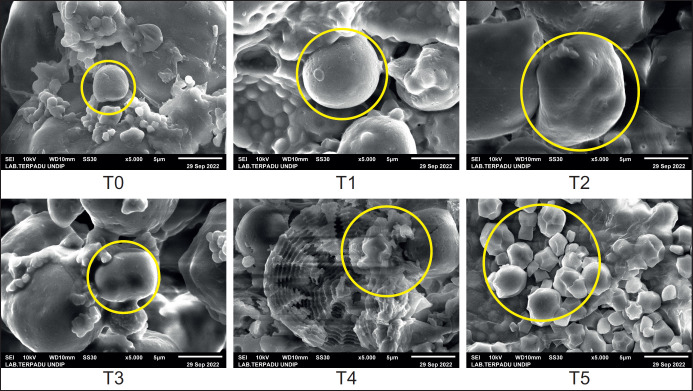
Observation of the composition of fodder *Sorghum* through SEM-EDX (5,000×).

The results of observing the composition of fodder *Sorghum* using SEM-EDX are shown in Table 3 and Figure 1.

Table 3 shows that the duration of planting SGF has an elemental composition of carbon (C) ranging from 85.55% to 96.89% or dominates compared to other elements. The element C contained in SGF comes from the natural constituent components of *Sorghum* seeds. The source of these elements can come from protein, where the protein content of SGF in the study was 9.43%–10.17%. The accumulation of *Sorghum* seed protein is influenced by carbon and nitrogen metabolisms because both depend on each other. Mrid et al. [24] stated that providing carbon skeletons for amino acids determines the protein content of grains such as cereals, where carbon and nitrogen metabolism depend on each other. Furthermore, the element C in *Sorghum* seeds comes from the plant’s ability to absorb nutrients from the soil through the roots. Rad et al. [25] stated that *Sorghum* is a type of legume that can live in warm/dry climates and can absorb nutrients from various soil levels as well as fix nitrogen (increase nitrogen content) in the soil, thereby increasing protein in seeds and forage. The duration of planting did not show changes in the composition of C elements in a certain direction, so it can be said that different planting times did not affect the SGF.

**Table 3.** Composition of fodder *Sorghum* using SEM-EDX.

Elemental composition	Treatment					
	T0	T1	T2	T3	T4	T5
	__________ % __________					
C	91.48	85.55	93.91	96.89	89.39	94.00
K_2_O	–	1.15	0.60	0.04	0.48	0.28
MgO	–	1.03	–	–	0.49	0.06
SO_3_	–	1.78	–	–	–	–
P_2_O_5_	–	1.91	–	–	–	–
ZrO_2_	8.52	8.58	5.49	3.07	9.63	5.67

The elemental compositions of potassium oxide (K_2_O), magnesium oxide (MgO), sulfur trioxide (SO_3_), and phosphorus pentaoxide (P_2_O_5_) had different compositions in each treatment, except for T0, which was not found at all. Elements of K_2_O and MgO in SGF ranged between 0.28%–1.15% and 0.06%–1.03%. Longer planting gives the *Sorghum* seeds time to form sprouts that can be a source of potassium and magnesium in fodder. The elements SO_3_ and P_2_O_5_ were only found in the T1 treatment at 1.78% and 1.91%, but the amounts were not too significant with other treatments. Elements of zirconium dioxide (ZrO_2_) in SGF amounted to 3.07%–9.63%. The element ZrO_2_ is obtained from rocks or the earth’s crust, which can be absorbed by *Sorghum* because of its ability to live in various soil conditions, so the element is also found in *Sorghum *seeds and, subsequently, in SGF. Joshi et al. [26] stated that the element ZrO_2_ could be useful as an antifungal agent for *Aspergillus fumigatus* with a maximum inhibition zone of 34 mm, *Aspergillus niger* (32 mm), and antibacterial *Bacillus subtilis* (36 mm), *Escherichia coli* (34 mm), *Pseudomonas aeruginosa* (32 mm), and *Streptococcus mutans* (28 mm).

The study results in Figure 1 show that different planting times affect the SEM image of fodder *Sorghum*. The SEM image of the SGF at T0 looks like it is still in the form of small and fine particles. The particles then enlarge with increasing duration of implantation until T4. The particle size again decreased at the time of T5 planting but by a higher amount than T0. The increasing particle size is an indication of the germination of *Sorghum* seeds into sprouts that have better nutritional content because they are at their maximum condition, so T4 treatment is the most recommended. Figure T4 shows a larger number of particles with a larger size. This is also supported by the data from Table 2 for nutritional content, where the T4 treatment had better protein than other treatments. Fodder constituent cells multiply and divide, as shown in the T5 treatment.

## Conclusion

SGF is considered suitable as a feed ingredient for poultry in terms of nutrition and contains ZrO_2_, which functions as an antifungal.

## List of Abbreviations

C, Carbon; EM, Energy metabolism; FTIR, Fourier transform Infrared Spectroscopy; K_2_O, Potassium oxide; MgO, Magnesium oxide; NFE, Nitrogen-free extract; SEM-EDX, Scanning electron microscope energy dispersive X-ray; SGF, Sorghum fodder; SO_3_, Sulfur trioxide; P_2_O_5_, Phosphorus pentoxide; ZrO_2_, Zirconium dioxide.
